# β_2_-Adrenogenic signaling regulates NNK-induced pancreatic cancer progression via upregulation of HIF-1α

**DOI:** 10.18632/oncotarget.5677

**Published:** 2015-10-16

**Authors:** Dong Zhang, Jianjun Lei, Jiguang Ma, Xin Chen, Liang Sheng, Zhengdong Jiang, Ligang Nan, Qinhong Xu, Wanxing Duan, Zheng Wang, Xuqi Li, Zheng Wu, Erxi Wu, Qingyong Ma, Xiongwei Huo

**Affiliations:** ^1^ Department of Hepatobiliary Surgery, First Affiliated Hospital of Xi'an Jiaotong University, Xi'an 710061, China; ^2^ Department of Oncology, First Affiliated Hospital of Xi'an Jiaotong University, Xi'an 710061, China; ^3^ Department of General Surgery, First Affiliated Hospital of Xi'an Jiaotong University, Xi'an 710061, China; ^4^ Department of Neurosurgery, Baylor Scott and White Health, Temple, TX, 76508, USA

**Keywords:** 4-(methylnitrosamino)-1-(3-pyridyl)-1-butanone, smoking, β2-adrenogenic signaling, HIF-1α, pancreatic cancer

## Abstract

Cigarette smoking is a risk factor for pancreatic cancer. It is suggested that 4-(methylnitrosamino)-1-(3-pyridyl)-1-butanone (NNK), a tobacco-specific nitrosamine, mediates the carcinogenic action of cigarette smoking by promoting cancer growth. In the present study, we show that smoking, HIF-1α expression and β_2_-adrenogenic receptor (β_2_-AR) expression are negatively correlated with the overall survival of pancreatic cancer patients. Moreover, HIF-1α expression and β_2_-AR expression are positively correlated with smoking status, different histological differentiation and among the tumor node metastasis (TNM) stages in pancreatic cancer patients. NNK increases HIF-1α expression in pancreatic cancer *in vitro* and *in vivo*. Furthermore, knockdown of HIF-1α and ICI118, 551 (a β_2_-AR selective antagonist) abrogates NNK-induced pancreatic cancer proliferation and invasion *in vitro* and inhibits NNK-induced pancreatic cancer growth *in vivo*. However, using CoCl_2_ (a HIF-1α stabilizing agent which decreases HIF-1α degradation under normoxia conditions) reverses ICI118, 551 induced effects under NNK exposure. Thus, our data indicate that β_2_-AR signaling regulates NNK-induced pancreatic cancer progression via upregulation of HIF-1α. Taken together, β_2_-AR signaling and HIF-1α may represent promising therapeutic targets for preventing smoking induced pancreatic cancer progression.

## INTRODUCTION

With a 5-year survival rate of less than 6% and more than 37,000 deaths per year, pancreatic ductal adenocarcinoma (PDAC) represents one of the most lethal human cancers and is the fourth leading cause of cancer-related death in the United States [1]. Increasing evidence suggests that many factors such as smoking, stress, and chronic depression may contribute to PDAC genesis and development, but the underlying mechanisms remain poorly understood [2–4]. The carcinogen NNK is formed from nicotine by nitrosation during the processing of tobacco in the mammalian organism [5]. Metabolites formed from NNK are strong mutagens that induce activating point mutations in k-Ras and inactivating mutations in p53 [6], both of which are common in human lung cancer and PDAC [7–10]. Previous studies indicate that smoking-stimulated NNK production and stress-stimulated activation of the autonomic nervous system promote tumor progression [11, 12]. Activation of the autonomic nervous system results in the release of catecholamines from the adrenal gland and sympathetic nerve terminals. Further studies suggest that both NNK and constitutive high levels of catecholamines modulate the activity of multiple components of the tumor microenvironment and consequently promote tumor cell growth via β-AR [4, 13–15].

β-AR are members of the superfamily of G protein-coupled adrenergic receptors, which mediate the actions of the endogenous catecholamines in a variety of target cells [16, 17]. β_1_- and β_2_-AR have been found to be expressed in the BxPC-3, MIA PaCa-2, and Panc-1 cell lines [18–20]. NNK functions as a β-AR agonist to induce cancer progression *in vitro* [21]. It has been shown that the binding of NNK to the β-ARs induce pancreatic cancer cell proliferation by activating the cyclic adenosine monophosphate (cAMP)/protein kinase A (PKA) pathways in pancreatic cancer cells [22]. The consequence of PKA signaling leads to the transcriptional activation of proteins involved in proliferation via cAMP response element binding protein (CREB), activator protein-1 (AP-1) or nuclear factor ‘kappa-light-chain-enhancer' of activated B-cells (NF-κB) [22–24]. We recently showed that β_2_-AR inhibitor ICI 118, 551 or HIF-1α inhibitor 2-methoxyestradiol could significantly inhibit the pancreatic cancer growth and angiogenesis induced by chronic stress, which suggested a novel β_2_-AR-HIF-1α regulatory axis for stress-induced pancreatic tumor growth and angiogenesis [25]. Moreover, activation of β-AR receptor could up-regulate levels of HIF-1α and downstream target genes independently of oxygen level in pancreatic cancer cells [26].

In this study, we focus on elucidating the role of HIF-1α and β_2_-AR signaling in NNK induced proliferation and invasion processes *in vitro* and *in vivo*. We show that NNK increases pancreatic cancer proliferation and invasion *in vitro* and promote pancreatic cancer growth *in vivo*. Knockdown of HIF-1α and ICI118, 551 abolishes the above effects of NNK. And CoCl_2_ increases ICI118, 551 suppressed pancreatic cancer invasion and cyclin D1 and VEGF expression under NNK exposure. Our data indicate that β_2_-AR signaling mediates NNK-induced pancreatic cancer progression via upregulation of HIF-1α.

## RESULTS

### Association of HIF-1α and β_2_-AR expression levels and smoking with pancreatic cancer

**Figure 1:**
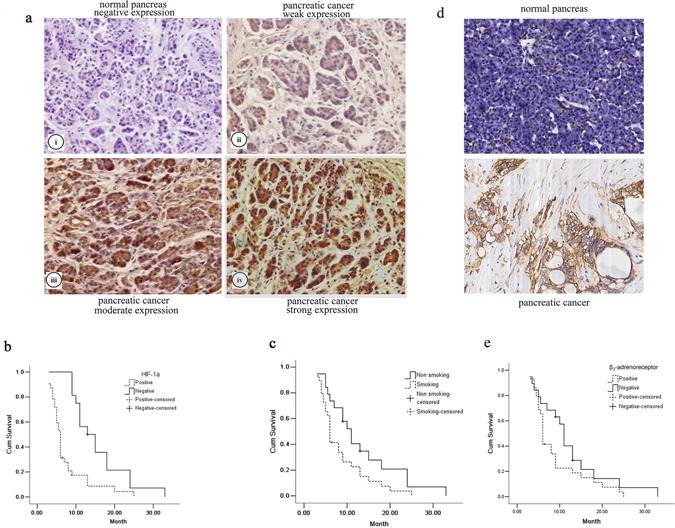
The effect of HIF-1α and β_2_-AR expression and smoking on pancreatic cancers **a.** Immunohistochemistry staining results showed HIF-1α expression (×200) on the normal tissues (i) and pancreatic cancer tissues (ii–iv): negative expression (i), weak expression (ii), moderate expression (iii) and strong expression (iv); Kaplan–Meier curves showed that **b.** the median survival time of the HIF-1α-positive and HIF-1α-negative group was 6.0 and 13.0 months, and **c.** the median survival time of the smoking-positive and smoking-negative group was 6.0 and 11.0 months, respectively. **d.** Immunohistochemistry staining results showed β_2_-AR expression (×200) on the normal tissues and pancreatic cancer tissues. **e.** Kaplan–Meier curves showed that the median survival time of the β_2_-AR-positive and β_2_-AR -negative group was 6.0 and 12.0 months.

To explore the possible role of HIF-1α and β_2_-AR in the triggering pancreatic cancer progression, we first explored the expression of HIF-1α and β_2_-AR in pancreatic cancer specimens. The immunohistochemistry staining showed that HIF-1α expression was not detected in normal pancreatic cells but was present in the cytoplasm of most pancreatic cancer cells. Moreover, β_2_-AR expression was hardly expressed in normal pancreatic cells but was present in the membrane of most pancreatic cancer cells. The representative staining results are shown in Figure 1a and 1d. Varying HIF-1α expression and β_2_-AR expression were observed between different histological differentiation and among the tumor node metastasis (TNM) stages (*P* < 0.05, respectively) (Table 1). The median survival time of the HIF-1α-positive and HIF-1α-negative groups were 6.0 and 13.0 months, respectively (*P* < 0.05) (Figure 1b). The median survival time of the β_2_-AR-positive and β_2_-AR-negative groups were 6.0 and 12.0 months, respectively (*P* < 0.05) (Figure 1e).

**Table 1:** The relationship between HIF-1α expression and clinical-pathologic character

Clinical and pathologic character	HIF-1α expression score	*P*-value	β_2_-AR expression score	*P*-value
Smoking (*n* = 29)	6.1	0.016	5.8	0.035
Non-smoking (*n* = 19)	3.8		3.5	
Pathology		0.020		0.024
Well-differentiated (*n* = 7)	2.9		2.5	
Moderately differentiated (*n* = 23)	4.4		4.3	
Poorly differentiated (*n* = 18)	5.8		5.3	
TNM stage		0.005		< 0.001
I phase (*n* = 7)	2.1		1.5	
II phase (*n* = 9)	3.8		3.4	
III phase (*n* = 19)	5.5		5.9	
IV phase (*n* = 13)	7.6		7.9	

HIF-α: hypoxia inducible factor-1α β_2_-AR; β_2_-adrenoreceptor; TNM: tumor node metastasis

Moreover, the median survival time of the smoking and non-smoking groups were 6.0 and 11.0 months, respectively (*P* < 0.05) (Figure 1c). Different HIF-1α and β_2_-AR expression levels were observed between the smoking and non-smoking groups (*P* < 0.05) (Table 1).

### Establishment of stable HIF-1α knockdown

**Figure 2:**
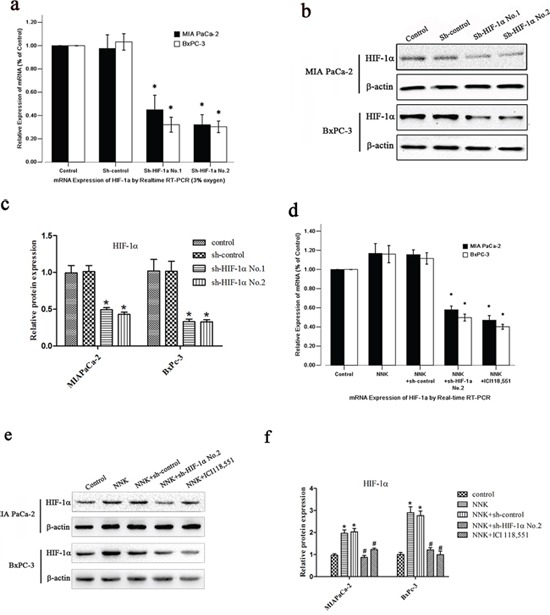
NNK induces HIF-1α protein upregulation in pancreatic cancer cells *in vitro* **a.** qRT-PCR analysis of HIF-1α expression after the transfection of different HIF-1α shRNA expression vectors under 3% oxygen exposure: sh-control, sh-HIF-1α No.1 and sh-HIF-1α No.2. All data were reported as the mean (± SD) of at least three separate experiments.*, *P* < 0.05, compared with sh-control cells. **b.** Western blot with HIF-1α antibody for different transfected pancreatic cancer cells under 3% oxygen exposure. β-actin was used as a loading control. **c.** Quantified analysis of Western blot results in b. *, *P* < 0.05 compared with sh-control group. **d, e**&**f.** After transfection, both BxPC-3 and MIA PaCa-2 cells were treated with NNK or ICI 118, 551. (d) qRT-PCR analysis of HIF-1α mRNA expression. All data were reported as the mean (± SD) of at least three separate experiments. *, *P* < 0.05 compared with NNK group. (e) Western blot analysis of HIF-1α protein expression. (f) Quantified analysis of Western blot results in e. *, *P* < 0.05 compared with control group. #, *P* < 0.05 compared with NNK group.

To study the long-term growth pattern of tumor cells *in vitro*, we successfully generated stable shRNA vector-transfected cells termed sh-HIF-1α No.1, sh-HIF-1α No.2 and sh-NC (negative control vector). qRT-PCR analysis showed that compared with sh-NC cells, the HIF-1α mRNA expression was inhibited up to 70% in both MIA PaCa-2 and BxPC-3 HIF-1α shRNA transfected cell lines under hypoxic conditions, particularly in sh-HIF-1α No.2 cells (*P* < 0.05). HIF-1α expression level in sh-NC cells was similar to that of normal cells (Figure 2a). Moreover, the HIF-1α protein level was also significantly downregulated in both MIA PaCa-2 and BxPC-3 HIF-1α shRNA transfected cell lines under hypoxic conditions (Figure 2b–2c).

### NNK increases HIF-1α protein expression in pancreatic cancer cells *in vitro*

Because different HIF-1α expression levels were observed between the smoking and non-smoking groups, we wanted to investigate the effect of NNK on the expression of HIF-1α *in vitro*. As shown in Figure 2d–2f, NNK markedly increased HIF-1α protein expression in both MIA PaCa-2 and BxPC-3 cells compared with the control group. However, HIF-1α mRNA expression in both MIA PaCa-2 and BxPC-3 cells remained unchanged.

Moreover, knockdown of HIF-1α abolished the effects of NNK on HIF-1α protein expression in both MIA PaCa-2 and BxPC-3 cells (Figure 2e–2f). Intriguingly, β_2_-AR antagonist (ICI 118, 551) also suppressed the HIF-1α protein expression induced by NNK (Figure 2e–2f).

### NNK enhances pancreatic cancer proliferation and invasion *in vitro* through β_2_-AR signaling

**Figure 3:**
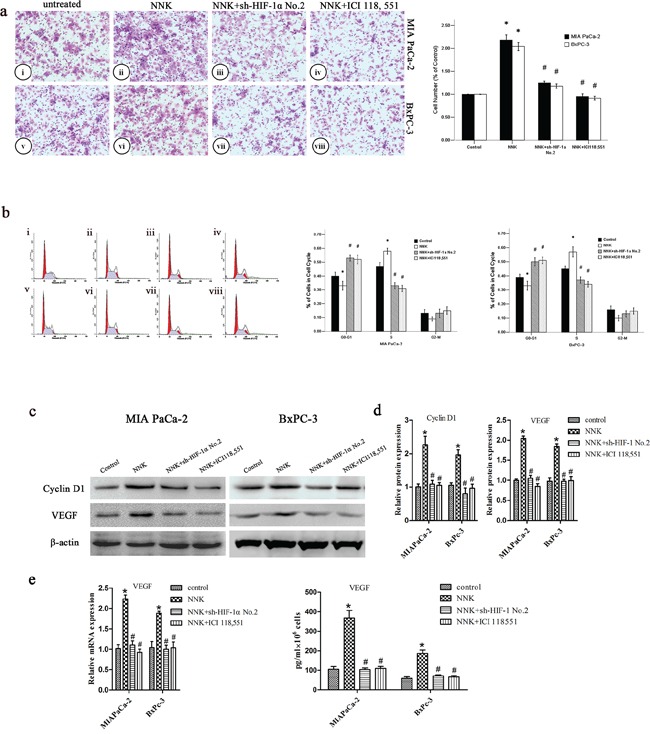
NNK promotes pancreatic cancer proliferation and invasion *in vitro* through β_2_-AR signaling **a.** Different group cells were seeded into a matrigel-coated invasion chamber for 24 h. The migrated cells were quantified by counting the number of cells in 10 random fields at × 100 magnification. MIA PaCa-2 cells were untreated (i) or treated: NNK (ii), NNK+sh-HIF-1α No.2 (iii), and NNK+ICI 118, 551 (iv) for 24 h. BxPC-3 cells were untreated (v) or treated: NNK (vi), NNK+ sh-HIF-1α No.2 (vii), and NNK+ICI 118, 551 (viii) for 24 h. All data were reported as the mean (± SD) of at least three separate experiments. *, *P* < 0.05 compared with control group. #, *P* < 0.05 compared with NNK group. **b.** Cell cycle distribution of different group cells was analyzed by FCM. MIA PaCa-2 were untreated (i) or treated: NNK (ii), NNK+sh-HIF-1α No.2 (iii), and NNK+ICI 118, 551 (iv) for 24 h, and BxPC-3 were untreated (v) or treated: NNK (vi), NNK+sh-HIF-1α No.2 (vii), and NNK+ICI 118, 551 (viii) for 24 h. All data were reported as the mean (± SD) of at least three separate experiments. *, *P* < 0.05 compared with control group. #, *P* < 0.05 compared with NNK group. **c.** Western blot assays were used to measure cyclin D1 and VEGF after different treatment of MIA PaCa-2 and BxPC-3 cells. **d.** Quantified analysis of Western blot results in c. *, *P* < 0.05 compared with control group. #, *P* < 0.05 compared with NNK group. **e.** qRT-PCR and ELISA analysis of VEGF expression after different treatment of MIA PaCa-2 and BxPC-3 cells. *, *P* < 0.05 compared with control group. #, *P* < 0.05 compared with NNK group.

NNK has been reported to be involved with colon or lung cancer progression through β_2_-AR signaling [15, 27]. In this study, we wanted to investigate whether NNK could modulate pancreatic cancer progression through β_2_-AR signaling. MIA PaCa-2 and BxPC-3 cells were treated with NNK. There was a marked increase in the invasive ability of both NNK-treated cell lines (Figure 3a). Moreover, we examined the effect of NNK on the cell cycle phase distribution of MIA PaCa-2 and BxPC-3 cells. We found a decrease in the proportion of cells in G0-G1 phase after treatment with NNK (Figure 3b), and an increase in the proportion of cells in S phase. In addition, cyclin D1 markedly increased after cells were exposed to NNK (Figure 3c–3e). We investigated whether NNK could upregulate the HIF-1α target genes. We found that expression of VEGF, a HIF-1α target gene, significantly increased after cells were treated with NNK, compared with VEGF expression in the control group (Figure 3c–3e).

However, incubation with the β_2_-AR antagonist (ICI 118, 551) abolished the effects of NNK in both MIA PaCa-2 and BxPC-3 cells (Figure 3). These data indicate that enhanced pancreatic cancer invasion and proliferation mediated by NNK are mainly dependent on β_2_-AR signaling *in vitro*.

### β_2_-AR signaling regulates NNK enhanced pancreatic cancer proliferation and invasion via upregulating HIF-1α *in vitro*

**Figure 4:**
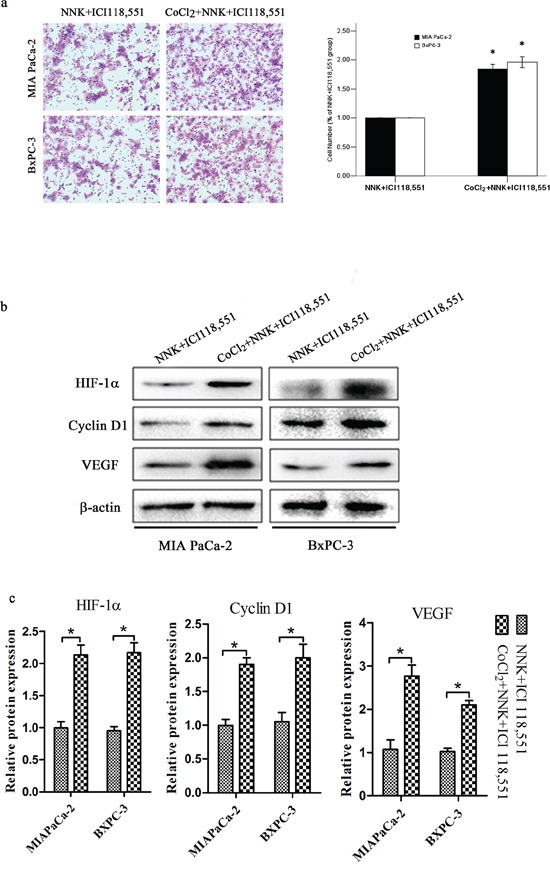
HIF-1α overexpression abolished β_2_-antagonist-induced effects on pancreatic cancer. Both BxPC-3 and MIAPaCa-2 cells were treated with 150 μM CoCl_2_ to induce HIF-1α overexpression **a.** Different group cells were seeded into a matrigel-coated invasion chamber for 24 h. The migrated cells were quantified by counting the number of cells in 10 random fields at × 100 magnification. All data were reported as the mean (± SD) of at least three separate experiments. *, *P* < 0.05 compared with NNK+ICI118, 551 group. **b.** Western blot assays were used to measure HIF-1α, cyclin D1 and VEGF after different treatment of MIA PaCa-2 and BxPC-3 cells. **c.** Quantified analysis of Western blot results in b. *, *P* < 0.05.

Next, we investigated whether HIF-1α contributes to NNK-induced pancreatic cancer proliferation and invasion. HIF-1α knockdown abrogates the effects of NNK on pancreatic cancer proliferation and invasion (Figure 3). Cyclin D1 and VEGF decreased significantly in the NNK+sh-HIF-1α group compared with the NNK group. Moreover, NNK treated MIAPaCa-2 and BxPC-3 cells were exposed to 150 μM CoCl_2_ to upregulate HIF-1α expression, and then, both two cells were treated with ICI 118, 551. The data showed that CoCl_2_ reversed the ICI118, 551 weakened invasion and significantly upregulated HIF-1α, cyclin D1, and VEGF expression in both two cells (Figure 4). Because the β_2_-AR receptor antagonist (ICI 118, 551) could suppress the HIF-1α protein expression and pancreatic cancer invasion and proliferation induced by NNK, all the results described above indicate that HIF-1α is required for β_2_-AR signaling mediating the NNK-enhanced pancreatic cancer invasion and proliferation *in vitro*.

### β_2_-AR signaling mediates NNK-induced tumor growth and VEGF expression *in vivo* via upregulation of HIF-1α

**Figure 5:**
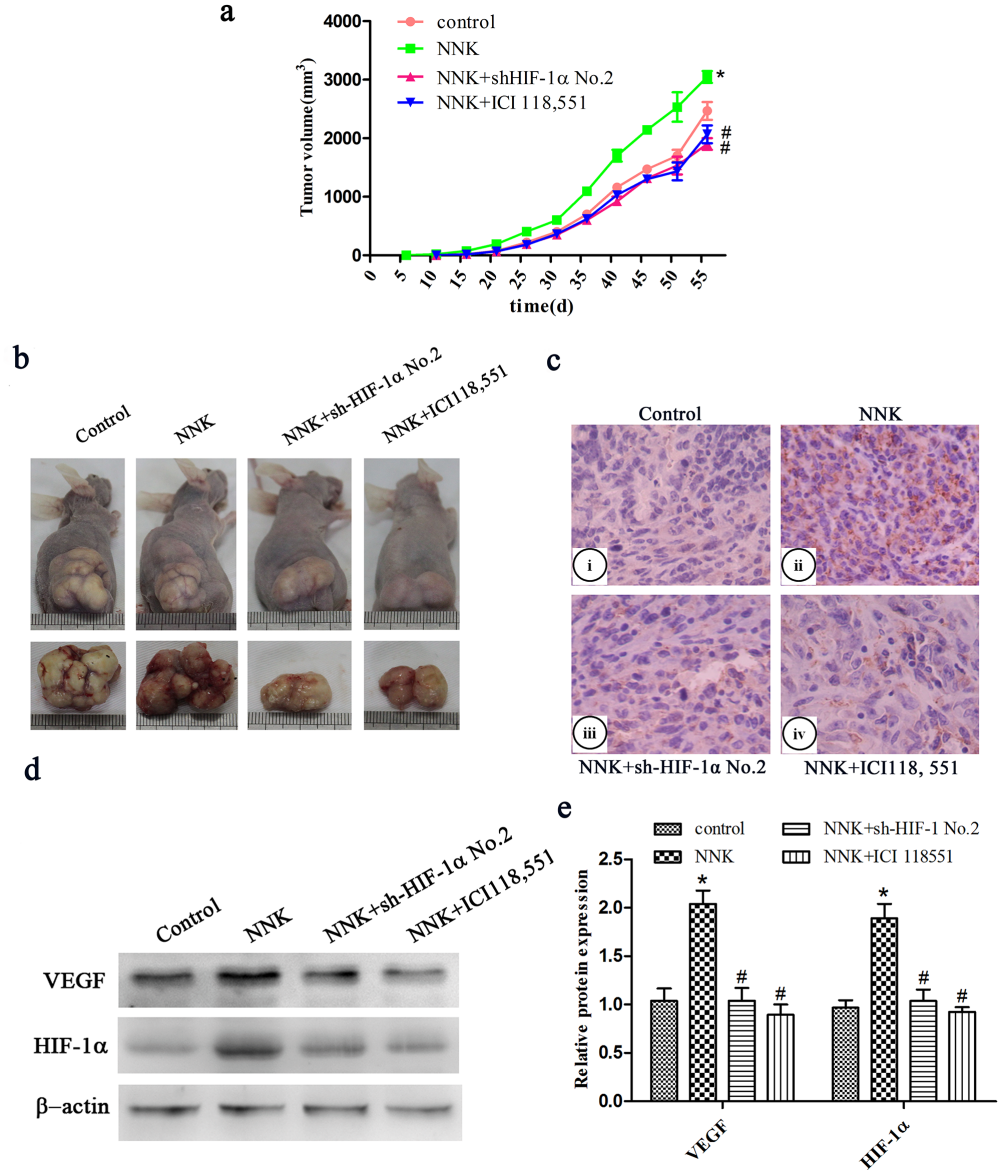
β_2_-AR signaling regulates NNK-induced tumor growth and VEGF expression *in vivo* through upregulation of HIF-1α Different group BxPC-3 cells were injected s.c. into the right flank of nude mice. **a.** Tumor growth curve of different group. **b.** Representative photograph of tumor sizes in different groups. **c.** Immunohistochemistry staining of VEGF expression in the xenografts. (i) Control group, (ii) NNK group, (iii) NNK+sh-HIF-1α No.2 group, (iv) NNK+ICI118, 551 group. **d.** Western blot analysis of HIF-1α and VEGF expression in the subcutaneous implant. **e.** Quantified analysis of Western blot results in c. *, *P* < 0.05 compared with control group. #, *P* < 0.05 compared with NNK group.

To further elucidate whether HIF-1α is also a mediator in β_2_-AR signaling regulating NNK-induced tumor growth and VEGF expression *in vivo*, stable HIF-1α knockdown BxPC-3 cells (sh-HIF-1α cells) and sh-NC cells were subcutaneously injected into nude mice. We found that NNK significantly increased tumor growth compared with the control group. Knockdown of HIF-1α or the β_2_-AR antagonist (ICI 118, 551) obviously restrained tumor growth compared with the NNK group (Figure 5a and 5b). Similarly, NNK significantly increased tumor VEGF expression. Additionally, abrogation of HIF-1α or ICI 118, 551 reversed the VEGF upregulation *in vivo* (Figure 5c–5e).

## DISCUSSION

NNK is a highly carcinogenic tobacco-specific nitrosamine but its role in smoking-related pancreatic cancer carcinogenesis remains unclear. In this study, we showed that NNK enhanced pancreatic cancer proliferation and invasion and the effect was abolished by a β_2_-antagonist. Treatment with NNK also increased the intracellular HIF-α level in MIA PaCa-2 and BxPC-3 cells. All these findings suggest that β_2_-ARs mediates the mitogenic action of NNK via a functional signal transduction pathway. While *in vitro* studies showed that NNK can stimulate PDAC cells directly by binding as an agonist to their β_2_-ARs [19, 23], the observed HIF-1α response adds a novel aspect to the mechanisms of action of this powerful tobacco carcinogen. These findings are in accord with reports on the significant stimulating effects of HIF-1α on the proliferation and metastasis of adenocarcinoma of the PDAC [28, 29], stomach [30], colon, prostate [31], and ovary [13, 14]. Accordingly, the NNK-induced increase in HIF-1α may contribute to the development of these cited tumors in smokers.

The findings described above clearly indicate that β_2_-ARs play an important role in the progression of smoking related pancreatic cancer. Our data suggest that the activation of β_2_-ARs would functionally result in increased cell proliferation, invasion accompanied with the up-regulation of HIF-1α. We recently showed that a HIF-1α-dependent β-AR signaling pathway mediated chronic stress-induced pancreatic cancer growth [25]. These findings might partially explain the protective effects of antioxidants in the treatment and prevention of pancreatic cancer in which multiple extracellular mitogenic signals have been reported to converge downstream of HIF-1α [32, 33]. More noteworthy is that treating MIA PaCa-2 and BxPC-3 cells with the β_2_-antagonist significantly downregulated the NNK increased HIF-1α expression along with the concomitant inhibition of cell proliferation and invasion, and HIF-1α overexpression abolished β_2_-antagonist-induced effects on pancreatic cancer. Our data indicate that β_2_-AR signaling regulates NNK enhanced pancreatic cancer proliferation and invasion via HIF-1α upregulation.

Smoking is a documented risk factor [34–36] and smokers have a 2-fold risk of developing PDAC [37, 38]. In this study, we were able to show that the median survival time of pancreatic cancer patients who are smokers is much shorter than that of patients who are non-smokers (6.0 and 11.0 months, respectively). Moreover, pancreatic cancer cells in smokers express higher levels of HIF-1α than in non-smokers, and high HIF-1α expression is correlated with low degree of histological differentiation and high TNM stages in human pancreatic cancer. Previous studies have shown that high HIF-1α mRNA expression had a significant impact on survival. High HIF-1α expression had a sensitivity of 87.1% and a specificity of 55.6% for the diagnosis short (<6 months) versus long (6–60 months) survival [39]. The expression of HIF-1α was correlated with tumor characteristics, microvascular density (MVD) and survival [40–42]. These data indicate that smoking may impact pancreatic cancer clinical outcomes through upregulation of HIF-1α. This is a retrospective study, which bears some limitations. The number of cases included may not be large enough, and the follow-up period is relatively short. A further prospective, randomized study is necessary to compare smoking exposure in more cases with longer follow-up.

Activation of β-ARs has been reported to stimulate pulmonary and colon cancer cell growth as well as pancreatic carcinoma cell migration [19, 20, 25, 28, 43]. To date, there are at least three reports showing a negative relationship between the use of β-blockers and cancer risk [44–47]. In these studies, β-blockers rather than diuretics or calcium channel blockers for cardiovascular diseases reduced the cancer risk. High expression of β_1_- and β_2_-ARs was found to be correlated with a low degree of histological differentiation and high TNM stages in human pancreatic cancer, further substantiating the role of β-ARs in the carcinogenesis of pancreatic cancer. These findings along with our experimental data might shed new light on the understanding of the carcinogenic mechanism in smoking-related pancreatic cancer and possibly open up new chemoprophylactic and therapeutic avenues for the prevention and treatment of cancers.

## MATERIALS AND METHODS

### Cell lines and culture conditions

Human pancreatic cancer cell lines (MIA PaCa-2 and BxPC-3) were stored in the Department of Hepatobiliary Surgery, the First Affiliated Hospital of Xi'an Jiaotong University. The cells were cultured in DMEM (Invitrogen, Carlsbad, CA, USA) supplemented with 10% FBS (Invitrogen, Carlsbad, CA, USA), penicillin (100 U/ml) and streptomycin (0.1 mg/ml). To stimulate MIA PaCa or BxPC-3 cells, 100 μM ICI 118, 551 (Sigma Chemical, St. Louis, MO, USA) or 100 μM NNK (Chemsyn Science Laboratories, Lenexa, KS) was added into the culture media.

### Immunohistochemistry

Patients diagnosed as pancreatic adenocarcinoma, with R0 resection, age 50–70y were included. Samples including 48 pancreatic carcinoma and 8 normal pancreas specimens were obtained from the Department of Hepatobiliary Surgery, the First Affiliated Hospital of Xi'an Jiaotong University. Immunohistochemical staining for HIF-1α and β_2_-AR were performed using the SABC kit (Maxim, Fuzhou, China) according to the manufacturer's instructions. Primary antibody for HIF-1α (1:50) and β_2_-AR (1:50) were obtained from Bioworld (St. Louis Park, MN, USA) and incubation occurred overnight at 4°C. Smokers are defined as patients who smoke cigarettes 20 pipe/day and last for 10 years. Non-smoking is defined as patients who never smoke. For the evaluation of protein expression, the staining intensity was graded as 0 (negative), 1 (weak), 2 (medium), or 3 (strong). The extent of staining was graded as 0 (0%), 1 (1–10%), 2 (11–50%), 3 (51–80%) and 4 (>81%) according to the percentage of positive staining area relative to the total tumor area. The final immunohistochemical staining score was obtained by multiplying the intensity and the extent of staining [48]. In the survival analysis, 48 patients diagnosed as pancreatic adenocarcinoma, with R0 resection. Among the total patients, 32 cases were detected positive expression of HIF-1α by immunohistochemistry, 16 cases were detected negative expression of HIF-1α. However, 3 cases were lost, 2 cases were from the positive HIF-1α expression group, and 1 case was from the negative HIF-1α expression group. Additionally, 29 patients were smokers, and 19 patients were non-smokers. Among the total 48 patients, the 3 cases were lost, 1 case a was smoker and 2 cases were non-smokers.

Signed informed consents from all the patients were obtained. The study protocol and consent forms were approved by the relevant ethical committee of the First Affiliated Hospital of Medical College, Xi'an Jiaotong University, China.

### HIF-1α shRNA lentivirus transfection

HIF-1α shRNA (shHIF-1α) and negative control shRNA (shNC) in eukaryotic GV248 lentiviral vectors were purchased from GeneChem Co., Ltd (Shanghai, China). The target sequence for HIF-1α shRNA was CCACCACUGAUGAAUUAAATT. Cells were seeded at 1 × 10^3^ cells/well into 96-well plates 24 h prior to transfection. When cells grew to 30–70% confluence, transfection was carried out by using lentiviral particles (MIA PaCa MOI = 20; BxPC-3 MOI = 10), polybrene (5 μg/ml) and ENi.S according to the manufacturer's protocol. 12 h post-transfection, virus-containing medium was replaced with complete medium. 96 h post-transfection, all cells were selected by puromycin (Merck, USA) at a final concentration of 5 μg/ml (MIA PaCa-2) or 4 μg/ml (BxPC-3) for 10 days. Then, cells were maintained in 2.5 μg/ml (MIA PaCa-2) or 2 μg/ml (BxPC-3) of puromycin. For generation of stable transfected cells, a hundred transfected cells were seeded into a 10 cm^2^ petri dish and media was changed three times a week. After 3 weeks, puromycin resistant colonies were isolated and seeded into 96-well plates for further study.

### Quantitative real-time polymerase chain reaction (qRT-PCR)

To extract total RNA, 2 × 10^5^ cells were harvested with the Trizol Reagent (Invitrogen, Carlsbad, CA, USA). cDNA synthesis was conducted as follows with the SYBR ExScript RT-PCR kit (Takara Biotechnology Co. Ltd., Dalian, China) according to manufacturer's instruction: 500 ng total RNA was mixed with 2 μl of 5× ExScript RTase buffer, 0.5 μl of dNTP mixture, 0.5 μl of random hexamers, 0.25 μl of ExScript RTase, and 0.25 μl of RNase inhibitor in a total volume of 10 μl. The reactions were performed at 42°C for 12 min, followed by inactivation of the reverse transcriptase at 95°C for 2 min. The cDNA was stored at 20°C. qRT-PCR was performed on an ABI PRISM 7300 Sequence Detection System (Applied Biosystems, Foster City, CA, USA) with the SYBR Green Master Mix. The final reaction volume was 25 μl and contained 12.5 μl 2× SYBR Premix Ex Taq, 1.0 μl of each primer (10 μM), 0.5 μl 50× ROX reference dye, and 1.0 μl cDNA. The cycling conditions were as follows: initial denaturation at 95°C for 10 min, followed by 40 cycles consisting of denaturation at 95°C for 5 sec, annealing at 60°C for 30 sec, and extension at 72°C for 30 sec. A melting curve analysis was applied to assess the specificity of the amplified PCR products. Each measurement was performed in triplicate, and no-template controls were included for each assay. GAPDH was applied as the internal housekeeping gene control. Relative gene expression was calculated using the 2^−ΔΔCt^ method [49]. The primers for HIF-1α were 5′-AAGTCTAGGGATGCAGCA-3′(forward) and 5′-CAAGATCACCAGCATCATG-3′ (backward). The primers for GAPDH were 5′-ACCACAGTCCATGCCATCAC-3′ (forward) and 5′-TCCACCACCCTGTTGCTGAT-3′ (backward).

### Flow cytometric analysis

Before flow cytometric analysis, 1 × 10^6^ cells were collected, washed two times with PBS, and fixed with ice-cold 70% ethanol for 24 h at 4°C. The fixed cells were stained with propidium iodide (Beckman Coulter, Miami FL, USA). After incubation for 30 min at 37°C, the samples were analyzed by Flow Cytometry. Cell cycle analysis of DNA histograms was performed with the MultiCycle software.

### Protein extraction and Western blot assay

MIA PaCa or BxPC-3 cells (1 × 10^6^) grown under our experimental conditions were lysed for 20 min on ice in 300 μl of RIPA lysis buffer (50 mM Tris-HCl (pH 7.5), 150 mM NaCl, 1% Triton X-100, 2 mM EDTA, 1 mM sodium orthovanadate, 1 mM phenylmethanesulfonyl-fluoride, 10 μg/ml aprotinin, 10 μg/ml leupeptin). Total proteins (100 μg) were loaded onto SDS-PAGE gels, separated, and transferred onto PVDF membranes (Roche, Penzberg, Germany). The membranes were blocked with 5% non-fat dry milk in TBST (10 mM Tris-HCl (pH 8.0), 150 mM NaCl, 0.05% Tween 20) and were subsequently incubated with primary antibodies overnight at 4°C. After five washes of 10 min each in TBST, the membranes were incubated with HRP-conjugated secondary antibodies for 2 h and subsequently washed again. The peroxidase reaction was performed using an enhanced chemiluminescence detection system to visualize the immunoreactive bands. The density of specific protein bands were determined by Image-Pro Plus 5.0 software (Media Cybernetics, Inc. Rockville, MD, USA).

### Matrigel invasion assay

A chamber-based invasion assay (Millipore, Billerica, MA, USA) was performed to evaluate pancreatic cancer cell invasion. Briefly, the upper surface of the membrane was coated with matrigel (BD Biosciences, Franklin Lakes, NJ, USA). MIA PaCa or BxPC-3 cells (1 × 10^5^) were resuspended in the upper chamber in serum-free medium and allowed to migrate toward a serum gradient (10%) in the lower chamber for 24 h. The media was aspirated from the inside of the insert, and the non-invasive cells on the upper side were removed by scraping with a cotton swab. The membrane was fixed with 4% paraformaldehyde and stained with crystal violet. The number of migrating cells was counted on each membrane in 10 random fields and photographed at × 100 magnification. The values reported here are the averages of triplicate experiments.

### Enzyme-linked immunosorbent assay (ELISA)

The cells were conditioned in serum-free medium for 72 h. The culture media were then collected and centrifuged at 1500 rpm for 5 min to remove particles. The supernatants were then frozen at −80°C until use. The production of VEGF in the supernatants of MIA PaCa or BxPC-3 cells was assessed by ELISA using a commercially available ELISA kit (R&D Systems, Minneapolis, MN, USA) according to the manufacturer's recommendations.

### *In vivo* tumor model

Female nude mice were purchased from Silaike Laboratory Animal Co., Ltd, Shanghai, China. The mice were housed and maintained under specific pathogen-free conditions in facilities approved by the Animal Care and Use Committee guidelines of the Xi'an Jiao Tong University, Shaanxi, China. Investigation has been conducted in accordance with the ethical standards and according to the Declaration of Helsinki and according to national and international guidelines and has been approved by the authors' institutional review board. The mice were used according to institutional guidelines when they were 6 to 8 weeks of age. Cells were resuspended in a 1:1 (v/v) mixture of culture media and Matrigel (BD Biosciences, San Jose, CA, USA), and 2 × 10^6^ BxPC-3 cells were injected s.c. into the right flank of nude mice. A total of five mice per group were used. Starting 24 hours after tumor cell injection, a 5 mg/kg/dose of ICI 118, 551 or PBS was i.p. injected into the mice every day, and NNK was i.p. injected into the mice three times a week for 8 weeks (50 mg/kg). All treatments were administered in a total volume of 200 μl. After 56 days, animals were sacrificed and the s.c. tumors were isolated. Tumors were fixed in formalin as soon as possible and embedded in paraffin. Tumor volume was calculated as (length/2) × (width^2^). Tumor samples were analyzed using H&E staining. Representative images were taken from each tumor using a light microscope at × 200 magnification.

### Statistical analysis

All statistical analyses were performed using the SPSS13.0 software. The results were presented as the means ± standard deviations (SD) of three replicate assays. Differences between the groups were assessed by Student's *t*-test or one way analysis of variance (ANOVA). *P* < 0.05 was considered as statistical significance.
